# Reduced brain activation in violent adolescents during response inhibition

**DOI:** 10.1038/srep21318

**Published:** 2016-02-18

**Authors:** Yi Qiao, Yi Mei, XiaoXia Du, Bin Xie, Yang Shao

**Affiliations:** 1 Shanghai Mental Health Center, Shanghai Jiao Tong University School of Medicine, Shanghai 200030, China; 2 East China Normal University, 3663 Zhongshan bei road, Shanghai 200062, China

## Abstract

Deficits in inhibitory control have been linked to aggression and violent behaviour. This study aimed to observe whether violent adolescents show different brain activation patterns during response inhibition and to ascertain the roles these brain regions play. A self-report method and modified overt aggression scale (MOAS) were used to evaluate violent behaviour. Functional magnetic resonance imaging was performed in 22 violent adolescents and 17 matched healthy subjects aged 12 to 18 years. While scanning, a go/no-go task was performed. Between-group comparisons revealed that activation in the bilateral middle and superior temporal gyrus, hippocampus, and right orbitofrontal area (BA11) regions were significantly reduced in the violent group compared with the control group. Meanwhile, the violent group had more widespread activation in the prefrontal cortex than that observed in the control group. Activation of the prefrontal cortex in the violent group was widespread but lacking in focus, failing to produce intensive activation in some functionally related regions during response inhibition.

Violence committed by adolescents is common throughout the world, with youths committing a range of crimes that cause death, injury, and disability^1^. The prevalence of deaths related to adolescent violence throughout the world is high^2^.

Impulsivity is a multidimensional concept that encompasses failure of response inhibition, rapid processing of information, novelty seeking, and inability to delay gratification^3^. Poor impulse control correlates with violent and aggressive behaviour^4^.

Response inhibition, the suppression of an inappropriate response, is one of the inhibitory executive functions that can be assessed by a go/no-go task. Go/no-go is a task that requires participants to respond to the target and refrain from responding to another target. Violent offenders have been shown to have impairments in inhibitory cognitive control^5^. LeMarquand’s study indicated that aggressive male adolescents made more errors compared to the nonaggressive group in a go/no-go task^6^, whereas another study found that the performance of the impulsive violent offenders was impaired only in the time pressure condition, which suggested that impulsive violent behaviour may be linked to abnormal modulation of the frontal cortical areas^7^.

At the neural level, some researchers found that the areas associated with aggressive and/or violent behavioural histories, particularly impulsive acts, were located in the prefrontal cortex and the medial temporal region after reviewing 17 neuroimaging studies^8^. Roberto and his colleagues conducted functional magnetic resonance imaging in a go/no-go task and found a positive correlation between motor impulsivity and activation of the bilateral ventrolateral prefrontal cortex during successful inhibitions in 24 healthy volunteers^9^. Many studies were conducted among psychiatric patients. A study conducted in mentally disordered violent individuals found both violent groups showed reduced thalamic activity, compared with controls, in association with modulation of inhibition by task demands^10^. Völlm conducted fMRI while performing a go/no-go task in borderline or antisocial personality disorder inpatients and found that in the healthy control group the main focus of activation during response inhibition was in the prefrontal cortex, specifically the right dorsolateral and left orbitofrontal cortex. Active regions in the patient group showed a more bilateral and extended pattern of activation across the medial, superior and inferior frontal gyri extending to the anterior cingulate^11^. Only a few studies have used fMRI to explore response inhibition deviations among adolescents with violent behaviour. A recent study measured response inhibition using a stop task in boys with conduct disorder and found that they had reduced activation in bilateral temporal parietal regions^12^. However, our understanding of response inhibition in violent adolescents remains poor.

The key novel aspect of the present study is the direct investigation of the relationship between trait impulsivity measures and brain activation during go/no-go tasks in adolescents with violent behaviours. Based on the previous studies, we hypothesized that 1) the violent adolescents would show abnormal activation in the prefrontal cortex as most studies have found; and 2) violent adolescents might have higher impulsivity, and this higher impulsivity would influence performance during the go/no-go task.

## Materials and Methods

### Subjects

Violent behaviour was defined with the two following methods in this study: 1) the modified overt aggression scale (MOAS)^13^,^14^ >4, and 2) at least 2 instances of violent behaviour in the past 6 months by self-report. The MOAS contains four subscales that represent verbal aggression, aggression against objects, physical aggression against oneself, and physical aggression against other people. Scores range from 0 to 4. The total MOAS score was obtained by assigning a different weight to each subscale score. This scale has good psychometric properties including reliability and validity^15^.

We screened violent adolescents in two reformatory schools and one vocational school. Nonviolent healthy adolescents were enrolled from a middle school, reported no violent behaviour in the past 6 months and had MOAS scores of less than 4. A total of 22 violent adolescents and 17 control adolescents aged from 12 to 18 years were enrolled in the study. All of the subjects were Chinese born, male, had normal or adjusted-to-normal visual acuity and were right-handed. The Barratt Impulsivity Scale-11 (BIS-11)^16^,^17^ was used to assess the impulsiveness of the participants (Table 1). The BIS-11 is one of the most widely used self-administered questionnaires of trait impulsivity with 30 items scored on a 4-point scale. It assesses long-term patterns of impulsive behaviours^16^. The three subscales describe motor, attention and non-planning impulsiveness. The methods were carried out in accordance with approved guidelines. All experimental protocols were approved by the Institutional Review Board of Shanghai Mental Health Center. Informed consent was obtained from all subjects.

### Go/no-go task

This go/no-go task required participants to press a button as quickly as possible after seeing an English letter displayed on a screen in front of them (go) and to refrain from responding to the letter “V” by not pressing the button (no-go). This task consisted of eight blocks. Each block contained 26 letters displayed for 0.5 seconds each. Between each letter, a one second interval displayed a black cross.

**Figure 1:**
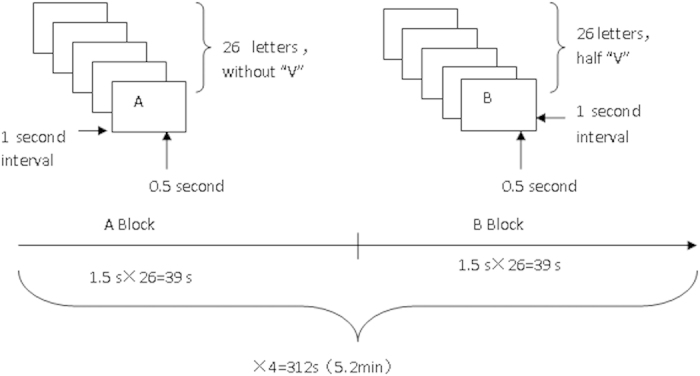
Design of the go/no-go tasks.

Two different types of blocks were presented: block A and block B. Block A contained all the letters except “V”, meaning participants needed to respond to all the letters in block A. Block B consisted of half “V” and half other letters requiring participants to respond to 50% of the letters by pressing the button. Block A and block B alternated 4 times during the task. Therefore, 75% of the targets were go, and 25% were no-go across the whole task (Fig. 1).

### Image acquisition

Images were acquired on a 3T system utilizing a head coil (Siemens, Germany). Time-to-repetition (TR) was 3,000 msec, time-to-echo (TE) was 30 msec, flip angle (FA) was 90 °, field of view (FOV) was 22 cm × 22 cm, matrix was 64 × 64 and slice thickness was 3 mm. The dist. factor was 33%, and the slice number was 32 (interleaved) with 104 volumes, 13 volumes for each block.

### Data analysis for brain imaging data

Image data were analysed with SPM5 (http://www.fil.ion.ucl.ac.uk/spm/). After removal of the first five scans, adjustments for timing differences in multi-slice image acquisition were performed. Next, realignments were made to correct for head movements. Subjects whose head movements exceeded 2 mm or 2 ° were excluded from the data pool. The data were spatially realigned to correct for personal differences and smoothed with an 8 mm^3^ kernel to remove the influence of noise. A general linear model was applied to estimate the activation figure. One-sample *t*-test was used to obtain the inner-group graph, and two-sample *t*-test was applied to the activation graph between groups. The activated brain regions were derived by Minispace. The brain map used by SPM is the International Consortium for Brain Mapping (ICBM) 152 human standard brain atlas from the Montreal Neurological Institute. SPSS 16.0 was used to perform the chi-square test and independent sample *t*-test.

## Results

### Behaviour and BIS-11 results

We calculated the errors or the number of times the participants pressed the button when the letter “V” was shown on the screen. An independent sample t-test was performed. The number of erroneous responses was 12.41 ± 5.33 (5.97%) in the violent group and 6.88 ± 4.18 (3.31%) in the control group during the test (*P* = 0.001). The response time to all targets (including right and wrong responses) averaged 394.82 ± 43.32 ms in the control group and 372.61 ± 46.34 ms in the violent group (P = 0.14).

Average scores for the total BIS-11, the non-planning factor, the motor factor and the attention factor were 68.14 ± 7.58, 23.0 ± 2.25, 26.27 ± 4.28 and 18.86 ± 2.97 in the violent group, respectively. The corresponding scores in the control group were 63.53 ± 7.68, 21.76 ± 3.29, 24.88 ± 3.69 and 16.88 ± 2.87, respectively. The P values were 0.70, 0.08, 0.80 and 0.67, respectively (see Table 1).

The Pearson correlation coefficients between the number of erroneous responses and total scores on the BIS-11 was 0.32 (P < 0.05), whereas the correlation coefficients with the non-planning factor, the motor factor and the attention factor were 0.32 (P < 0.05), 0.31 (*P* > 0.05) and 0.08 (P > 0.05), respectively. The correlation coefficient between response time and total BIS-11 score was −0.03 (P > 0.05), whereas the correlations with the non-planning factor, the motor factor and the attention factor were −0.10 (P > 0.05), 0.01 (*P* > 0.05) and 0.08 (P > 0.05), respectively.

### fMRI results

#### Within-group analysis

**Figure 2:**
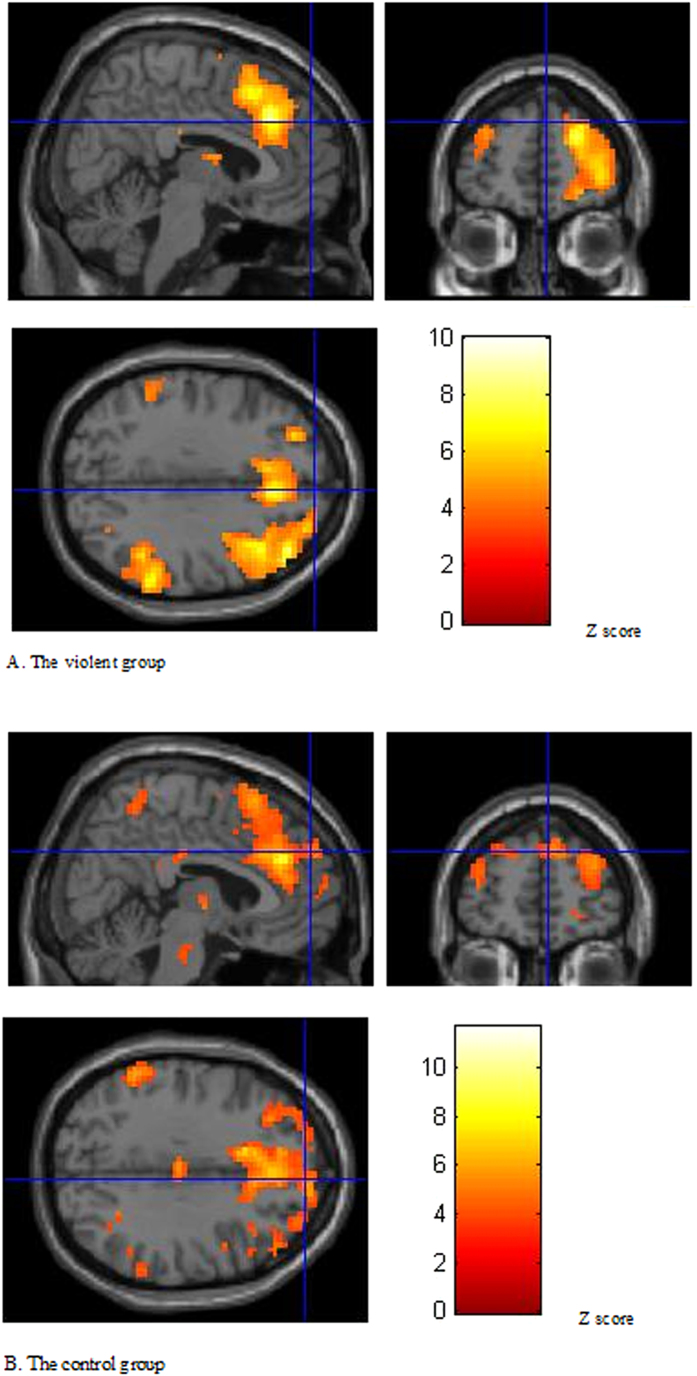
Brain activation of no-go minus go. The crossing site is BA 9.

The threshold for activation was set at *p* < 0.001. In the no-go condition compared to the go condition, the right middle frontal gyrus, bilateral insula, right middle temporal gyrus, left inferior parietal lobule, supramarginal gyrus and bilateral cingulate gyrus were detected to be activated in the violent group. In the control group, the activated areas included the bilateral middle temporal gyrus, superior temporal gyrus, right inferior frontal gyrus, left supramarginal gyrus, right superior parietal lobule, cingulate cortex and left body of the caudate nucleus (Tables 2 and 3, Fig. 2).

#### The activated volume in the region of interest (ROI)

As reported by a previous study^8^, activation in the prefrontal cortex correlates with aggression and violent behaviour. Therefore, we set the region of interest in this area. The violent group’s activation was more extensive in the bilateral prefrontal cortex than that observed in the control group (146.83 ± 99.52 mm^3^ vs 72.58 ± 50.91 mm^3^, *t* = 2.30, P < 0.05) when comparing the No-go condition to the go condition (Table 4).

#### Between-group comparisons

**Figure 3:**
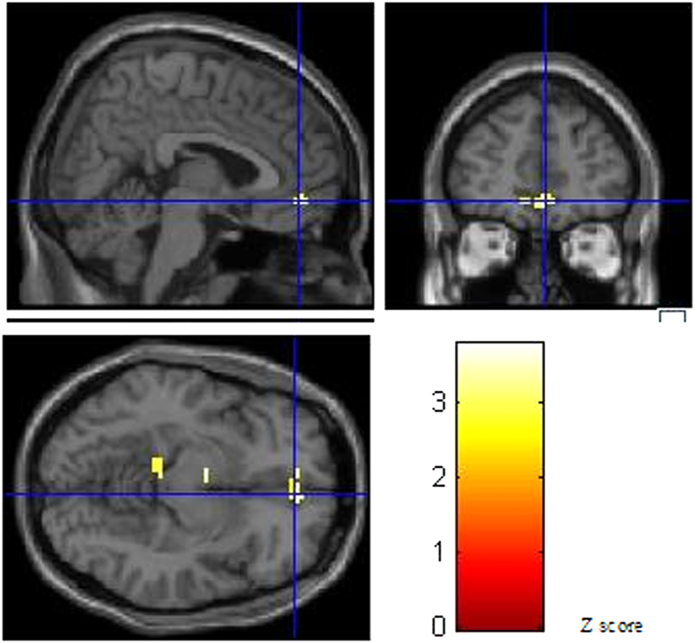
Images of prefrontal activation (control group minus violent group, coordinate: x = 4.93, y = 46.73, z = −9, BA11).

Between-group comparisons revealed that activation in the bilateral middle temporal gyrus, superior temporal gyrus, hippocampus, and right orbitofrontal area (BA11) regions were significantly reduced in the violent group compared to the control group (Fig. 3). The analyses were made after controlling for the go task at an uncorrected threshold of *P*  <  0.05.

#### The correlation between BIS-11 and brain activation

We performed correlation analyses between the BIS-11 scale and the brain activation regions where significant differences were found between groups. Thus, the bilateral middle and superior temporal gyrus, hippocampus, and right orbitofrontal areas were included. The Pearson correlation coefficients was −0.18 between the total score on the BIS-11 and average brain activation of those three regions mentioned before (P > 0.05). We also performed a correlation analysis between the BIS-11 and the bilateral middle and superior temporal gyrus, hippocampus, and right orbitofrontal areas separately. The correlation coefficients were −0.17, −0.23 and −0.01, respectively (P > 0.05). There were no correlations between average brain activation and scores on the subscales. The correlation coefficients between average brain activation and the non-planning factor, the motor factor and the attention factor were −0.10, −0.15 and −0.17, respectively (P > 0.05).

## Discussion

Though the score on the total BIS-11 and the subscales did not differ between the violent group and the control group, the erroneous responses made by the violent group during the go/no-go task were much higher than the control group. Moreover, the erroneous responses had positive correlations with the total BIS-11 score (*R* = 0.32, P < 0.05) and the non-planning factor (*R* = 0.32, P < 0.05) in this study. The violent group did show higher impulsivity, especial non-planning impulsivity, than the control group in the current study on a trend level, but the difference was not statistically significant (P = 0.08). This indicated that impulsivity might not necessarily be high in adolescents with violent behaviours, but it led to more mistakes when response inhibition was needed. Higher impulsivity, especially non-planning impulsivity, might cause more errors on a behavioural level. In two previous studies, researchers found there was no significant correlation between impulsivity and task performance, but those studies were conducted with healthy volunteers^9^,^18^.

Previous neuroimaging studies have shown that the dysfunctional brain areas associated with aggressive and/or violent behavioural histories are located in the prefrontal cortex and medial temporal regions^8^. We expected to observe differences in these areas. In this study, the violent group was more extensively activated in the prefrontal cortex than the control group. This finding was similar to that of Vollum who worked with patients with antisocial and borderline personality disorders^11^. Meanwhile, between-group comparisons showed that the right orbitofrontal area of the violent group was less activated than the control group. Aron found that the patients with right frontal lobe lesions responded slower on the stop signal reaction time task - a sensitive estimate of inhibitory control^19^. An fMRI study found that neural response during response inhibition was most prominent in the right lateral orbitofrontal cortex in normal subjects^20^. Similarly, the current study found more activation in the right orbitofrontal area in the control group, indicating it may play an important role in the executive function of response inhibition. Although the violent group showed more extensive activation in the prefrontal cortex, the activation of the right orbitofrontal area was insufficient. From this, we speculate that activation of the prefrontal cortex of violent adolescents is more extensive but fails to produce intensive activation in some functionally related regions. We speculate that the prefrontal cortex network is not as well organized in violent adolescents as in control adolescents, and this might weaken response inhibition.

Between-group analysis also found that the bilateral middle temporal gyrus and superior temporal gyrus were less activated in the violent group during response inhibition. Some studies have suggested that the superior temporal sulcus normally provides the amygdala with visual information that contributes to the identification of the affective or motivational significance of visually perceived objects^21^. We speculate that the capacity to recognize and process the visual information is poorer in violent adolescents.

The hippocampus is a structure in the temporal cortex within the limbic system. There are correlations between the limbic system and antisocial pathology^22^. Previous research reported abnormal hippocampal volume in habitually violent offenders^23^, and strong negative correlations were observed between the psychopathic scores and the volume of the posterior half of the hippocampus bilaterally. In recent research, abnormal hippocampal shape was observed in violent offenders with psychopathy^24^. The bilateral hippocampus was less activated in the violent group in this study. This might suggest some functional defects of the hippocampus in violent individuals.

This study did not find a correlation between the BIS-11 and brain activation where significant differences were found between groups. The bilateral middle and superior temporal gyrus, hippocampus, and right orbitofrontal area were included. This indicated that impulsivity did not affect the brain activation in these areas significantly, but it might have had a negative correlation with the brain activation on a trend level.

Our findings support the hypothesis that the violent group would show less activation in the prefrontal cortex (right orbitofrontal area) and medial temporal regions during response inhibition. In addition, the bilateral superior temporal gyrus and hippocampus were less activated in the violent group. This indicates that these brain regions might have some dysfunctions in violent adolescents during response inhibition. We investigated the impulsivity of the participants and found that impulsivity might not necessarily be high in adolescents with violent behaviours, but it led to more mistakes when response inhibition was needed. Our findings also support the hypothesis that higher impulsivity, especial non-planning impulsivity, causes more errors on a behavioural level. However, we did not find a correlation between impulsivity and brain activation.

## Additional Information

**How to cite this article**: Qiao, Y. *et al.* Reduced brain activation in violent adolescents during response inhibition. *Sci. Rep.* **6**, 21318; doi: 10.1038/srep21318 (2016).

## Figures and Tables

**Table 1:** Demographic and behavioural characteristics of the violent group and the control group.

	Violent group (n = 22)	Control group (n = 17)	*P*
Age	15.59 ± 1.05	15.94 ± 1.25	0.40
Years of education	9.73 ± 1.16	10.18 ± 1.33	0.74
MOAS score	15.14 ± 3.24	0.59 ± 2.18	0.01
Number of violent episodes in past 6 months	6.32 ± 9.96	0	0.00
BIS-11 total score	68.14 ± 7.58	63.53 ± 7.68	0.70
Non-planning factor	23.0 ± 2.25	21.76 ± 3.29	0.08
Motor factor	26.27 ± 4.28	24.88 ± 3.69	0.80
Attention factor	18.86 ± 2.97	16.88 ± 2.87	0.67

**Table 2:** Brain areas showing significant activation in the violent group (no-go minus go).

L/R	Brodmann’s Area	MNI coordinate			Voxels	Z score
		x	y	z		
R	6/10	45	45	18	1644	6.92
R	32	42	3	45	337	6.48
L	10/47	−27	48	24	303	5.71
L	46	−45	42	21	32	5.34
R	13	33	21	3	48	6.53
L	13	−36	12	−12	55	5.79
R	7/21	54	−27	−15	571	6.37
L	40	−54	−45	42	533	4.82
L	23/24	−9	−24	36	51	3.60
R	33	3	−24	24	9	4.95
L	40	−54	−45	33	120	4.77

L/R: left/right, MNI coordinate: Montreal Neurological Institute coordinate, *P* < 0.001.

**Table 3:** Brain areas showing significant activation in the control group (no-go minus go).

L/R	Brodmann’sArea	MNI coordinate			Voxels	Z score
		x	y	Z		
R	21	54	−27	−12	337	6.04
R	21	63	−45	0	337	4.20
L	21	−63	−27	−12	58	4.73
L		−15	9	12	12	4.72
R	7	36	30	−66	54	4.22
R	47	36	21	−15	27	4.90
L	47	−30	15	−12	171	5.40
L	32	−12	24	36	80	5.37
R	32	6	36	27	79	5.29
M		0	−21	30	238	5.17
R	23	3	−36	24	10	3.59
R	38	36	6	−24	460	4.91
L	21/22	−60	−48	9	94	4.59
L	40	−54	−51	36	111	4.44

L/R: left/right, MNI coordinate: Montreal Neurological Institute coordinate, P < 0.001.

**Table 4:** The activated volume ROI (Prefrontal Cortex).

Brodmann area	9L	9R	10L	10R	6L	6R	8L	8R	46L	46R	47L	47R
Control group	92	155	22	93	55	152	21	111	0	27	60	83
Violent group	44	272	109	264	100	306	109	264	32	86	65	111

L/R: left/right, volume: voxel, unit: mm^3^.
